# How Does Integrated Care Do With, Not for, Neighbourhoods & Communities?

**DOI:** 10.5334/ijic.11104

**Published:** 2026-08-12

**Authors:** Robin Miller, Michelle L. A. Nelson, Fraser Battye

**Affiliations:** 1 University of Birmingham, United Kingdom; 2 School of Epidemiology and Public Health, University of Ottawa, Canada; 3 Strategy Unit, United Kingdom

**Keywords:** neighbourhoods, community, integrated care

## Abstract

A notable development in recent years has been the emphasis on structuring integrated care around ‘neighbourhoods’ or ‘places’ and engaging ‘communities’ in the planning, design and delivery of support. Across the world, related principles are being used to inform national and regional policies and to develop local practice innovations. Despite such interest, community and neighbourhood working can feel a radical endeavour which clashes with established structures, relationships, investments and skills. A group of international experts from policy, practice, communities and research came together at a roundtable at the International Conference on Integrated Care 2026 to debate how integrated care can authentically adopt these principles. Agreeing what is meant by such concepts and how they can be utilised in practice is an important starting point – ‘neighbourhoods’ were generally seen to be more geographically oriented and where people physically live, whereas ‘communities’ relate to the connections and relationships which matter to people. Addressing power and resource imbalances requires investing in the voluntary and community sector and framing neighbourhoods as the responsibility of the whole system, including hospitals. Building the professional skills and capacity for neighbourhood working means embedding as a core competency with qualifying and on-going education. Creating the organisational and system capacities to sustain involves developing performance metrics and processes which emphasis shared outcomes. Such developments also require researchers to support with the process of change through responsive, co-produced and accessible methodologies.

## Introduction

A notable development in recent years has been the emphasis on structuring integrated care around ‘neighbourhoods’ or ‘places’ and engaging ‘communities’ in the planning, design and delivery of support. Across the world, related principles are being used to inform national and regional policies and to develop local practice innovations [1,2,3]. Such ideas are of course not new. For example, in the past community health and social work professionals in Western countries were often based in local patches with time and flexibility to develop longer term connections with local people before the move to efficiency and process optimisation ruptured these relationships. General practitioners used to live in the same buildings as their practices and truly served their patients within the local area from cradle to grave. Within the Global South, community orientated approaches have been the norm within many countries [4,5].

There is now robust evidence that formal health services directly shape only a fraction of health outcomes. Estimates consistently suggest that hospitals and primary care collectively account for less than 20% (and some estimates are as low as 10%) of what determines health, with the remainder driven by social, economic, behavioural and environmental conditions that operate at the neighbourhood and community level [6]. This reframes neighbourhood working not as a desirable complement to clinical healthcare, but as necessary engagement with the core foundations of what enables wellbeing. And there is also growing evidence on the health effects of social connection which reinforces that the neighbourhood may be the smallest meaningful scale at which wellbeing-enabling conditions can be cultivated and sustained [7].

Despite such experience and evidence, community and neighbourhood working can feel a radical endeavour which clashes with established structures, relationships, investments and skills. Therefore, whilst there may be common agreement regarding this overall direction of travel, what it looks like in practice is often uncertain and contested. This is not the first moment of policy ambition for local, integrated, out-of-hospital care. Across multiple decades and health systems, the dominant pattern has been aspiration toward neighbourhood-oriented approaches followed by reversion to hospital-centric investment and delivery. The question, therefore, is not only whether such working is desirable, but what conditions might make this moment different.

Against this backdrop, a group of international experts from policy, practice, communities and research came together at a roundtable at the International Conference on Integrated Care 2026 to debate how integrated care can authentically adopt such principles. This perspective article shares the themes of this roundtable with key points being illustrated by quotes from the discussion.

## Define communities and neighbourhoods

The first step is agreeing what is meant by such concepts and how they can be utilised in practice – ‘neighbourhoods’ were generally seen to be more geographically oriented and where people physically live, whereas ‘communities’ relate to the connections and relationships which matter to people –

‘*strong ties are your community, weak ties are your neighbourhood*’

It is important to note that weak ties are not necessarily a lesser form of connection. Research consistently shows that casual contact with acquaintances contributes meaningfully to daily wellbeing and a sense of belonging, and for people who are socially isolated, these peripheral ties may be their primary source of social contact [8]. Therefore, the neighbourhood matters because proximity and routines generate ties to others. The physical environment plays a major role in supporting these connections through creating accessible, safe and affordable places in which people can interact.

Many communities can exist within and across neighbourhoods, and some communities, such as those who are homeless and travelling people, do not have a geographic basis at all. Neighbourhoods (and particularly those of a smaller scale) are often seen as a helpful way to structure health and care delivery, but this must be balanced with the recognition that people may work, learn and connect elsewhere. A good start for local integrated care initiatives is to identify the places which already act as a hub for local people – this could be a high street, a school, a library or a sports club, and building around the social connections these provide –

‘*it’s go wherever the footfall is and try and make that the integration space*’

## Address power and resource imbalances

An inclusive approach to engaging with communities should recognise their distinct histories, dynamics and traumas, and entails ‘creating welcoming and calming spaces for people and with people and deeply respecting lived experience’. Often the vehicle to achieve such spaces is through collaboration with voluntary and community sector (VCS) organisations, due to their trusted nature and deep connections with beneficiaries –

‘*instead of parachuting in, if it’s grown from the within the community, then it looks like the community, because it is the community*’

However, integrated care initiatives often fail to recognise the challenges that the VCS often faces in relation to their financial position and are unwilling or unable to address the investment disparities between the VCS and formal health and care services. To attain neighbourhood working within thriving, connected places integrated care must invest in radically different ways. Even when funding is tight, there is opportunity to take a systems view, and to more effectively bring together the resources currently tied up in different sectors and services to promote greater wellbeing –

‘*many of the levers sit out with health care and social care. It’s in education, it’s in economic regeneration, it’s in transport, lifelong learning, you name it. So the money is there but we’re just not tapping in*’

When additional funding is available, the process through which this is allocated can further emphasise the power differential between formal health and care and the VCS, and weaken collaborative relationships through requiring competitive behaviours between organisations –

‘*those types of relationships, they take years to build but take minutes to destroy*’

It is also important to resist locating responsibility for this change solely with neighbourhood-level organisations and professionals. Systemic barriers, hospital-centric incentive structures, workforce distribution, and capital investment decisions, are made and sustained at regional and national levels, and must be addressed there. Furthermore, the field has tended to focus on VCS organisations as the primary community partners, while underutilising the potential of anchor institutions. Hospitals, universities, and local government, are themselves significant neighbourhood actors as major employers, landowners, and generators of community footfall. Engaging them as partners in neighbourhood working is often an underexplored opportunity.

## Measure what matters

Whilst policies and practice guidelines are increasingly adopting the ‘language’ of communities and neighbourhoods, the performance and incentive frameworks in which professionals and services operate are slow to adopt their principles. This leads to a disconnect between the aspirations of integrated care leaders to be more community orientated and collaborative, and the realities of what measures will be used to decide if they are running ‘successful’ or ‘failing’ organisations and partnerships –

‘*chief execs will say, well, I’m going to be fired for not meeting my four-hour emergency department wait times, not because I’ve not been a good partner in driving for with the neighbourhood health approach*’

Along with this misalignment between measurement and outcomes, the burden of responding to burdensome performance processes can stifle the capacity and energy to work more creatively with community partners –

‘*as one chief officer said the other week in a public meeting, stop my staff having to fill up these spreadsheets weekly for stuff that actually nobody really cares about*’

Adding to these frustrations, in the long term it is those more preventative approaches which are being crowded out which will lead to improved health and wellbeing –

‘*services try to meet demand, but communities reduce demand*’

## Build skills, capacity and sustainability

Whilst reorganising services around neighbourhood is a helpful footprint, for practitioners to connect with communities requires knowledge and skills which have often been lost within health and social care. Such community engagement is under-represented in the curriculum of qualifying programmes and competency frameworks and are therefore no longer core to professional cultures –

‘*social care, lost the plot – we had to take social workers out into the communities. Did they know who the people were? Did they know the neighbourhood? Did they know who the members and shakers were? It’s just beyond belief how we had to reacquaint them with those basic things*’

A further barrier are hierarchies of status within professions in which becoming a clinical specialist has higher prestige than roles which are more holistic and relational. This diverts early career professionals to hospital-based disciplines, and is connected with results in investment disparities –

‘*this drives research, which supports innovation, that draws more money into acute care, and it’s a virtuous cycle or a vicious cycle, depending on which way you look at*’

A further challenge is sustainability. Most current examples of neighbourhood and community-oriented integrated care are innovations, pilots, or proof-of-concept initiatives. However, even when the initial signs are positive, becoming established practice requires embedding in governance, workforce planning, and organisational cultures. This requires investment and actions beyond the energy of individual champions. Building the skills and capacity for community and neighbourhood working must therefore include the organisational and system capacities needed to sustain it.

## Conclusion

Integrated care can and must better connect with where people live (their neighbourhood) and the connections that give them meaning and support (their communities). Doing so effectively, and ethically, requires a recognition of long-standing power imbalances between formal health and care services and the voluntary and community sector, and a willingness to take tough decisions to move substantial funding from existing delivery methods to build community capacity [9]. It also entails health and social care professionals accepting their limitations and being open to learning from those from other disciplinary backgrounds. Reorganising services to reflect natural neighbourhoods and centres of community will not by themselves lead to radical change in practice [10]. Neighbourhood approaches also carry an inherent risk of deepening inequality: more affluent, better-organised communities tend to benefit disproportionately from place-based initiatives, while those most in need may be least able to mobilise. Equity must therefore be an explicit design principle, not an assumed outcome.

Achieving these changes at the scale and speed required will need the shared sense of urgency experienced during the Covid pandemic. Embedding neighbourhood and community approaches can not be the sole responsibility of those who already work in those settings but must be a whole system approach supported practically and economically by hospital and specialist care. Such developments also require researchers to support with the process of change through responsive, co-produced and accessible methodologies [10]. Just how committed governments, services and professionals are to the difficult decisions and loss of institutionalised power connected with these worthy and necessary aspirations remains to be seen. As one of the roundtable participants framed it –

‘Neighbourhood working is not about engaging a diverse population: it’s about learning from and listening to people’.
